# Novel Variants of *ANO5* in Two Patients With Limb Girdle Muscular Dystrophy: Case Report

**DOI:** 10.3389/fneur.2022.868655

**Published:** 2022-04-08

**Authors:** Matthew Katz, Fleur C. Garton, Mark Davis, Robert D. Henderson, Pamela A. McCombe

**Affiliations:** ^1^Department of Neurology, Royal Brisbane and Women's Hospital, Brisbane, QLD, Australia; ^2^Institute for Molecular Bioscience, The University of Queensland, Brisbane, QLD, Australia; ^3^Department of Diagnostic Genomics, Pathwest Laboratory Medicine, Perth, WA, Australia; ^4^Centre for Clinical Research, The University of Queensland, Brisbane, QLD, Australia

**Keywords:** case report, limb girdle muscular dystrophy, ANO5, novel variant, next generation sequencing

## Abstract

Here we report on two unrelated adult patients presenting with Limb girdle muscular dystrophy who were found to have novel variants in *ANO5*. Both patients had prominent weakness of their proximal lower limbs with mild weakness of elbow flexion and markedly elevated creatine kinase. Next generation sequencing using a custom-designed neuromuscular panel was performed in both patients. In one patient, 336 genes were targeted for casual variants and in the other patient (using a later panel design), 464 genes were targeted. One patient was homozygous for a novel splice variant [c.294+5G>A; p.(Ala98Ins4^*^)] in *ANO5*. Another patient was compound heterozygous for two variants in *ANO5*; a common frameshift variant [c.191dupA; p.(Asn64fs)] and a novel missense variant [c.952G>C; p.(Ala318Pro)]. These findings support the utility of next generation sequencing in the diagnosis of patients presenting with a Limb girdle muscular dystrophy phenotype and extends the genotypic spectrum of *ANO5* disease.

## Introduction

Anoctamin 5 (ANO5) is a protein that belongs to a family of calcium-activated chloride channels and phospholipid scramblases (1). The exact function of the ANO5 protein is not completely understood although it has been shown to play a role in sarcolemma repair (1). Recessive variants of *ANO5* have been associated with a range of muscle diseases including limb girdle muscular dystrophy (LGMD R12), (2, 3) distal myopathy (Miyoshi-like muscular dystrophy type 3), (4) exercise induced myalgia, (5) recurrent rhabdomyolysis, (6) axial myopathy (7) and asymptomatic hyperCKemia (5).

Patients with LGMD R12 typically present in adulthood with slowly progressive proximal lower limb and bicep weakness that is often asymmetric. This is associated with markedly elevated creatine kinase (CK) levels (2, 3). For unknown reasons females homozygous for *ANO5* variants tend to have a less severe phenotype than males (5). Dilated cardiomyopathy and cardiac conduction disease have been reported but are rare (8). Magnetic resonance imaging (MRI) of the lower limbs often shows selective atrophy and fatty infiltration of the posterior calf and thigh muscles (9).

Here we report the clinical, pathologic, and genetic findings of two unrelated patients presenting with LGMD who were found to have novel variants in *ANO5*.

## Case Report

### Patient 1

This 35-year-old Iranian man presented with slowly progressive upper and lower limb weakness from the age of 20 years. At the time of presentation, the patient had difficulty standing from the seated position and washing his hair. There were no sensory symptoms. There was no significant medical history. The patient was borne to non-consanguineous parents. His brother had similar symptoms, but four other siblings were unaffected.

**Figure 1:**
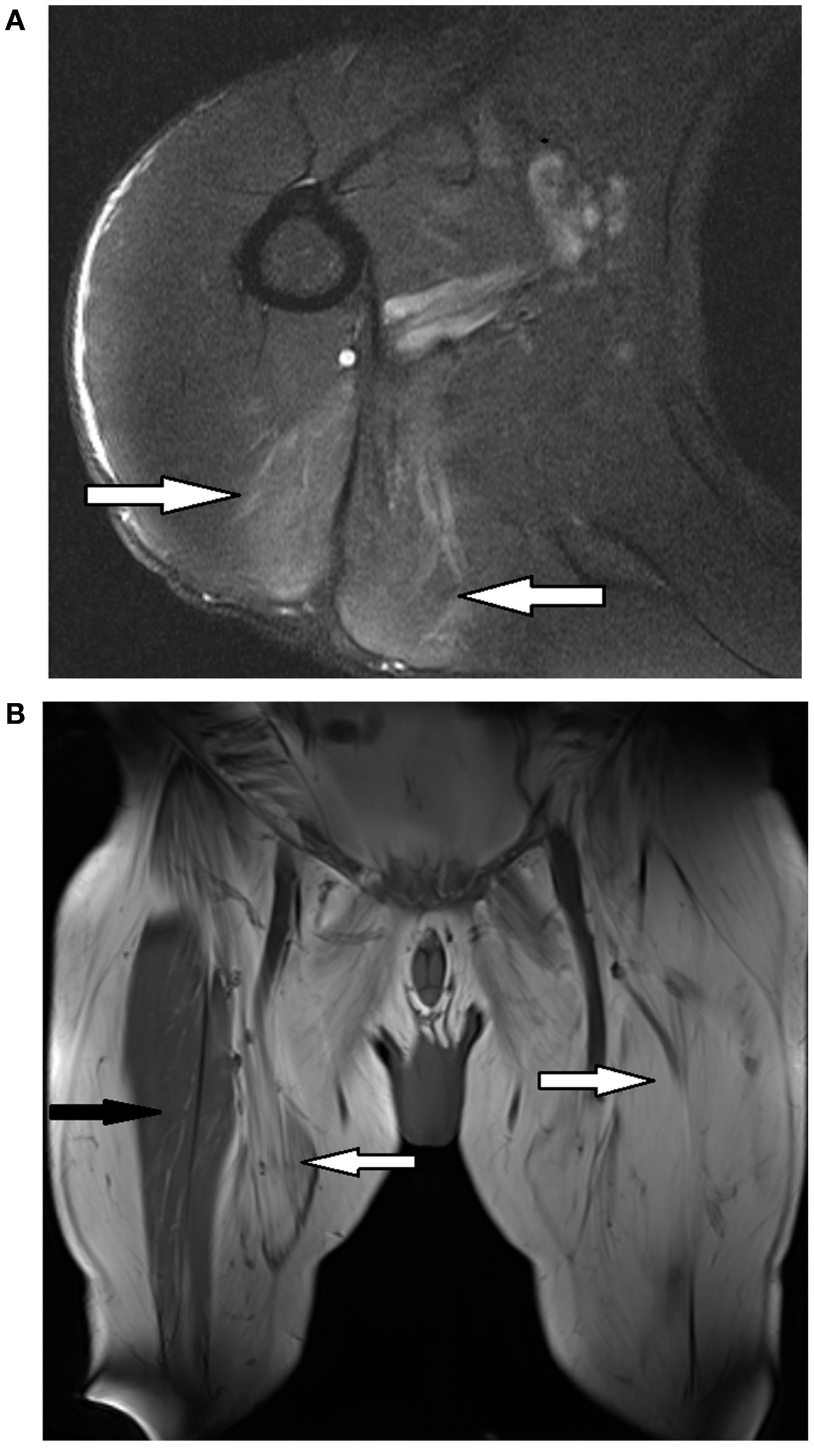
Magnetic resonance imaging (MRI) of selected muscles from patients 1 and 2. **(A)** Left shoulder of patient 1 - axial T2* weighted sequence showing increased T2 signal of teres major and long head triceps (white arrows). **(B)** Bilateral thighs of patient 2 - coronal T1^∧^ weighted sequence showing extensive fatty replacement of anterior and medial thigh muscles (white arrows) with relative preservation of the right rectus femoris (black arrow). *T2, transverse relaxation time; ^∧^T1, longitudinal relaxation time.

On examination he had a waddling gait. There was no muscle wasting. Tone was normal. There was MRC 4/5 weakness of hip flexion and extension, hip adduction and knee flexion and extension. There was MRC 4+/5 weakness of elbow flexion bilaterally. Distal upper and lower limb strength was normal (MRC 5/5). Facial, ocular and neck strength was normal (MRC 5/5). Reflexes were normal. The sensory examination was normal. The CK was 1990. An MRI of the upper limbs performed 3 years earlier showed increased T2 signal of teres major and long head of triceps bilaterally without atrophy or fatty infiltration (Figure 1). A muscle biopsy showed end-stage muscle disease consistent with a chronic muscular dystrophy.

Previous genetic testing for Becker muscular dystrophy and facioscapulohumeral muscular dystrophy (FSHD) was negative. The patient had also undergone targeted sequencing of the fukutin related protein (*FKRP*) gene, which was normal. Next generation sequencing (NGS) using a custom-designed 336 gene neuromuscular panel (PathWest Laboratory, Australia) (10) revealed homozygosity for a novel splice variant [c.294+5G>A; p.(Ala98Ins4^*^)] in intron 5 of the *ANO5* gene (ClinVar https://www.ncbi.nlm.nih.gov/clinvar/variation/1326848/?new_evidence=true).

**Figure 2:**
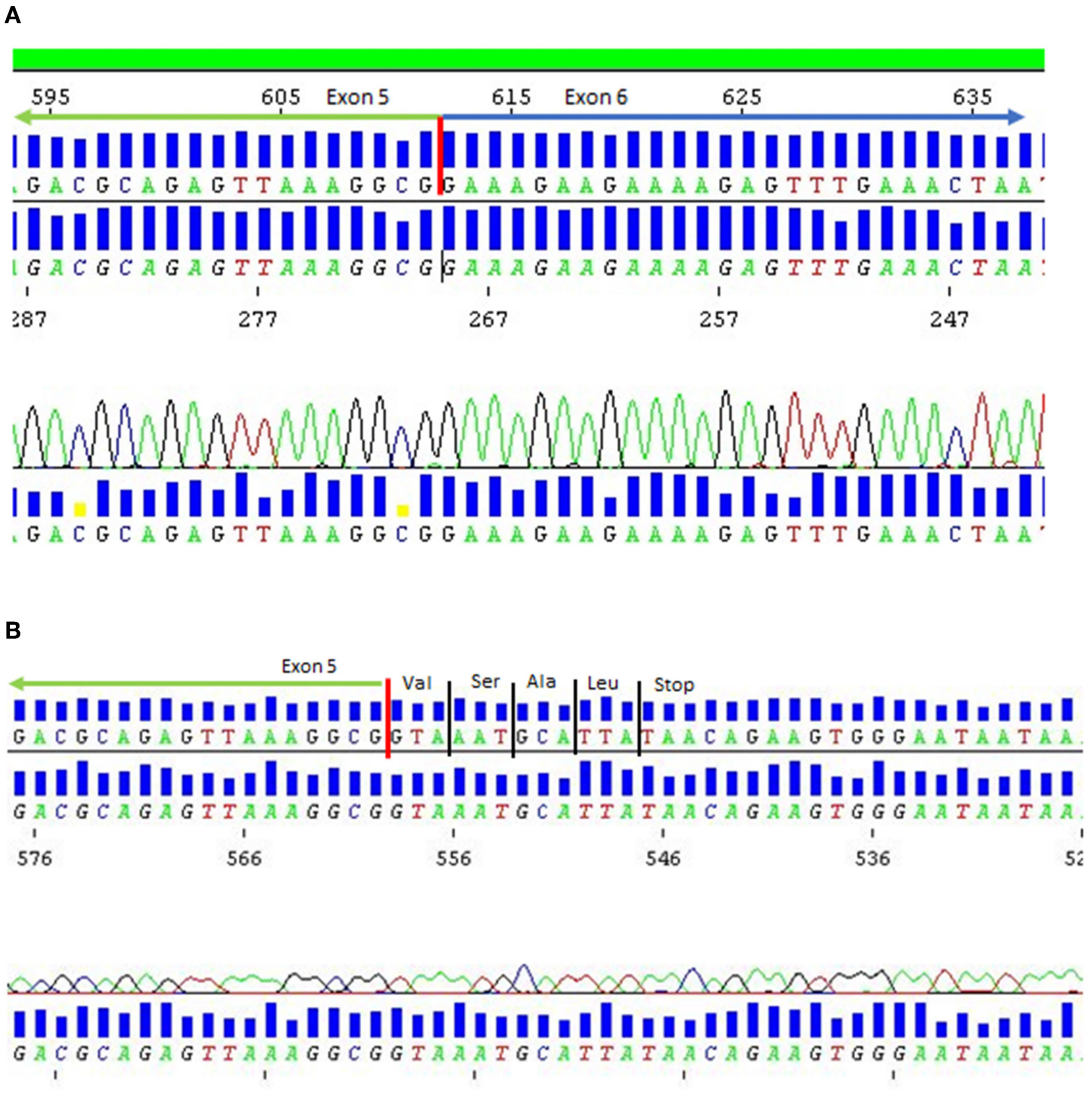
DNA sequence chromatogram for patient 1. **(A)** Normal control mRNA. **(B)** Patient 1 mRNA showing loss of exon 5 donor splice site resulting in intron inclusion and premature termination of protein due to presence of stop codon in variant mRNA.

While *in silico* analysis was not indicative of a likely splice effect, complementary DNA studies demonstrated intron inclusion and a complete absence of normal product (Figure 2). His brother was also found to be homozygous for the same variant on targeted sequencing of *ANO5*.

### Patient 2

This 55 year-old man presented with slowly progressive lower and upper limb weakness from the age of 35 years. At the time of presentation, he had difficulty rising from the seated position and walking up stairs. There was no significant medical history. There was no family history of muscle weakness.

On examination he had a waddling gait. There was wasting of the calf muscles bilaterally. The other limb muscles were of normal bulk. Tone was normal. There was MRC 4/5 weakness of hip flexion, hip extension, hip adduction, hip abduction, knee flexion and right knee extension. There was MRC 3/5 weakness of left knee extension. There was MRC 4+/5 weakness of elbow flexion and extension bilaterally. Distal upper and lower limb strength was normal (MRC 5/5). Facial, ocular and neck strength was normal (MRC 5/5). Reflexes were reduced in the upper and lower limbs. The sensory examination was normal. The CK was 4,910.

An EMG showed myopathic motor units without abnormal spontaneous activity in the right vastus medialis, vastus lateralis, biceps femoris and medial gastrocnemius. There were fibrillations with reduced recruitment of large motor units in the right tibialis anterior. MRI of the upper and lower limbs showed fatty infiltration and mild atrophy of the posterior calf and thigh muscles along with fatty infiltration and atrophy of the pectoral, latissimus dorsi and teres muscles (Figure 1). An echocardiogram did not show evidence of cardiomyopathy. The patient declined to undergo a muscle biopsy.

Next generation sequencing using a custom-designed 464 gene neuromuscular panel (PathWest Laboratory, Australia) (10) revealed compound heterozygosity for two variants in the *ANO5* gene; a previously reported pathogenic frameshift variant [c.191dupA; p.(Asn64fs)] in exon 5 that results in a premature stop signal and a novel missense variant [c.952G>C; p.(Ala318Pro)] in exon 10 (ClinVar https://www.ncbi.nlm.nih.gov/clinvar/variation/1327991/?new_evidence=false#id_first). Segregation studies were not possible as the patient's first-degree relatives were not available for genetic testing.

## Discussion

We describe two patients presenting with LGMD who were found to have novel *ANO5* variants after NGS using a custom-designed neuromuscular genetic panel. The use of multigene panels is especially helpful for the diagnosis of LGMD R12 as its phenotype can overlap with other types of LGMD (11) and muscle biopsies in these patients are often non-diagnostic and show non-specific myopathic or dystrophic changes (7, 12) although skeletal muscle amyloid deposits have been identified in some cases (13). The identification of disease causing variants with NGS also helps with genetic counseling in those with LGMD by allowing for targeted genetic testing in family members that could be carriers or at risk of inheriting the disease.

Patient 1 was a 35-year-old Iranian man who was homozygous for a novel splice variant [c.294+5G>A; p (Ala98Ins4^*^)]. This variant leads to intron inclusion resulting in premature truncation of the protein, is absent from controls (14–17) and segregates with disease in the affected sibling (13, 18) (Figure 2 and Table 1). Patient 1 has Iranian ancestry. Most reported cases of LGMD R12 have been in European patients (3). However, LGMD R12 has been reported in patients from the Middle East, including Saudi Arabia (18), Jordan (19) and Afghanistan (20).

**Table 1:** Genetic summary for *ANO5* variants.

**Patient**	**Transcript**	**hg38**	**Codon**	**Amino acid**	**POPmax**	***In silico***	**ACMG-AMP classification**
	**reference**		**variant**	**change**		prediction^*^	
Patient 1	NM_213599.3	11:22221215	c.294+5G>A	p.Ala98Ins4^*^	NR	–	Pathogenic (PVS1, PM2, PP1)
Patient 2	NM_213599.3	11:22250310	c.952G>C	p.Ala318Pro	NR	DDD	Variant of uncertain significance (PM2, PM3, PP3)
	NM_213599.3	11:22221107	c.191dupA	p.Asn64fs	0.0021	–	Pathogenic (PVS1, PS4, PP5)

*ACMG-AMP = American College of Medical Genetics and Genomics - Association for Molecular Pathology (version: 8.4.7 from Varsome), hg38 = human reference genome, NR = allele not previously reported, POPmax = maximum allele frequency in each reference population from 1000GP, ExAC, gnomAD, and ESP*.

* *= Meta SVM, Meta LR, and M-CAP in silico prediction (“D” = deleterious, “T” = tolerated)*.

Patient 2 was a 55-year-old Caucasian man who was compound heterozygous for the frameshift variant [c.191dupA; p.(Asn64fs)] and a novel missense variant [c.952G>C; p.(Ala318Pro)]. Through a founder effect, the frameshift variant [c.191dupA; p.(Asn64fs)] is the most common pathogenic *ANO5* variant identified in European patients with LGMD R12 (3). The missense variant [c.952G>C; p.(Ala318Pro)] in patient 2 is absent from controls, is strongly conserved (21) and predicted to be damaging *in silico* (22, 23) (Table 1). Currently considered a variant of uncertain significance as per the American College of Medical Genetics and Genomics—Association for Molecular Pathology (ACMG-AMP) guidelines (24) additional functional evidence could help elucidate its impact. Detailed, mutation-specific assessments across *ANO5* are lacking in the literature but loss of *ANO5* has been shown to cause defective membrane repair (1). The novel missense variant we report in patient 2 is located within the first transmembrane domain of the protein (25) and could influence the effectiveness of membrane transport for repair (ie. coordination of annexins), particularly in muscle where it is highly expressed (26). The clinical presentation of patient 2 with adult onset, slowly progressive proximal lower limb and biceps weakness and significantly elevated CK is typical of other cases of LGMD R12 reported in the literature (2, 3).

## Conclusion

*ANO5* variants are not uncommon in LGMD but occur with a spectrum of LGMD severity. Our findings extend the genotypic spectrum of *ANO5* disease and supports the utility of next generation sequencing in the diagnostic process. Further work on understanding disease mechanisms underlying *ANO5* associated LGMD is needed to address relevant therapeutic avenues.

## Data Availability Statement

The datasets presented in this article are not readily available because of ethical and privacy restrictions. Requests to access the datasets should be directed to the corresponding author/s.

## Ethics Statement

Ethical review and approval was not required for the study on human participants in accordance with the local legislation and institutional requirements. The patients/participants provided their written informed consent to participate in this study. Written informed consent was obtained from the individual(s) for the publication of any potentially identifiable images or data included in this article.

## Author Contributions

FG and MD assisted with the genetic data and review of paper. RH and PM assisted with overall review of the paper. All authors contributed to the article and approved the submitted version.

## Conflict of Interest

The authors declare that the research was conducted in the absence of any commercial or financial relationships that could be construed as a potential conflict of interest.

## Publisher's Note

All claims expressed in this article are solely those of the authors and do not necessarily represent those of their affiliated organizations, or those of the publisher, the editors and the reviewers. Any product that may be evaluated in this article, or claim that may be made by its manufacturer, is not guaranteed or endorsed by the publisher.
